# Analytical Validation of MyProstateScore 2.0—Active Surveillance: A Urinary-Based Clinical RT-PCR Prostate Cancer Assay

**DOI:** 10.3390/diagnostics16101486

**Published:** 2026-05-14

**Authors:** Tabea M. Setera, Cameron J. Seitz, Bradley S. Moore, John R. Kitchen, Spencer Heaton, Jingyi Cao, Jacob I. Meyers

**Affiliations:** Lynx Dx, Ann Arbor, MI 48108, USA; tabea@lynxdx.com (T.M.S.); cameron@lynxdx.com (C.J.S.); john@lynxdx.com (J.R.K.); jingyi.cao@lynxdx.com (J.C.)

**Keywords:** prostate cancer, MyProstateScore2.0—active surveillance, urinary biomarkers, active surveillance, analytical validation

## Abstract

**Background/Objectives:** Active surveillance (AS) is recommended for men with low-risk prostate cancer to minimize overtreatment while monitoring for disease progression. However, current surveillance strategies rely heavily on repeat biopsies, which are invasive and associated with morbidity. MyProstateScore 2.0—Active Surveillance (MPS2-AS) is a urine-based biomarker test developed to predict progression to Grade Group ≥ 2 (GG ≥ 2) and Grade Group ≥ 3 (GG ≥ 3) prostate cancers in men on AS. The objective of this study was to analytically validate the reproducibility and robustness of MPS2-AS analyte detection and risk score calculation across key laboratory variables. **Methods:** Analytical precision was evaluated using pooled urine specimens processed using the MPS2-AS laboratory workflow. Eight pooled urine samples were tested in a within-laboratory design across five days, with two runs per day, and two replicates per run. Additional reproducibility studies assessed variability across three QuantStudio™ 12K Flex Real-Time PCR Systems and three OpenArray™ chip lots. Ten RNA biomarkers were quantified by RT-PCR and used to calculate the MPS2-AS GG1-2 and GG1-3 risk scores. Variance components were estimated using hierarchical ANOVA. **Results:** The MPS2-AS analyte measurements demonstrated high precision across within-laboratory testing, with standard deviations ranging from 0.00 to 0.60 and coefficients of variation (%CV) from 0.00 to 4.01%. The reproducibility across qPCR instruments and OpenArray chip lots showed similar robustness, with analyte %CVs of ≤4.57% and ≤4.10%, respectively. These stable analyte measurements translated to reproducible model outputs, with %CV ≤ 10.69% for the GG1-2 risk score and ≤7.20% for the GG1-3 risk score across all tested conditions. No systematic bias was observed between runs, days, instruments, or reagent lots. **Conclusions:** MPS2-AS demonstrates strong analytical precision and reproducibility for quantifying urinary biomarkers and generating GG1-2 and GG1-3 risk scores. These results support the reliability of MPS2-AS for clinical laboratory implementation and its use as a non-invasive tool to inform biopsy decisions in men with Grade Group 1 prostate cancer undergoing active surveillance.

## 1. Introduction

Clinical guidelines recommend active surveillance (AS) as the management strategy for men with low-risk prostate cancer (PCa), seeking to reduce overtreatment of cancers that pose minimal clinical risks while monitoring for any cancer progression that would necessitate treatment [1,2]. Preference for this approach has grown and the rate of men opting into AS has increased substantially, with nearly 60% of patients with Grade Group 1 (GG1) disease now managed on AS [3,4].

AS programs incorporate serial prostate-specific antigen (PSA) testing, multiparametric magnetic resonance imaging (mpMRI), and prostate biopsies to monitor for disease progression [5,6]. Yet, each of these comes with important limitations. PSA kinetics do not reliably predict higher-grade cancer in AS patients, such that repeated invasive biopsies have remained a necessary component of monitoring [7]. While mpMRI adds diagnostic value, it has been shown to miss an unacceptably high 41% of higher-grade cancers [8]. Moreover, MRI is resource dependent, and its population-wide use is limited by availability; dependence on expert interpretation; and, in the United States, cost [9,10,11]. As a result, repeat biopsies remain an important component of AS, but are associated with pain, infections, bleeding, and urinary complications, with the morbidity risk increasing with each subsequent biopsy [12,13,14]. Negative biopsy experiences often result in patients unsafely delaying or dropping off from AS monitoring [15,16,17,18].

Accurate, non-invasive biomarker tests have the potential to reduce the burden of invasive monitoring by assessing cancer progression risk and helping to determine whether a repeat biopsy is warranted for men on active surveillance. Yet, no biomarker test has demonstrated clinically suitable performance [19]. MyProstateScore 2.0—Active Surveillance (MPS2-AS) is a urine-based test that builds on the established biomarker foundation of the commercial MyProstateScore 2.0 (MPS2) test, which was validated in biopsy-naïve and prior negative biopsy populations to predict risk of GG ≥ 2 cancer [20,21,22,23]. Following the same laboratory protocol as MPS2 to amplify a subset of the MPS2 biomarker panel, MPS2-AS predicts individualized risks of GG≥2 and GG≥3 cancers for men on active surveillance. Separate predictions of GG≥2 and GG≥3 progressions support shared decision-making, where some GG2 progression may be monitored with continued surveillance and routine biopsy schedules, whereas a GG≥3 risk more strongly supports expedited confirmatory or surveillance biopsy [5,24]. In addition to providing risk scores for GG≥2 and GG≥3 cancers, MPS2-AS risk stratifies men into low-risk and increasing-risk categories. At the validated threshold of 11.5%, low-risk MPS2-AS results rule out progressions to GG≥2 and GG≥3 with sensitivities of 95% and 97% and NPVs of 92% and 99% respectively, allowing physicians and patients to reasonably defer biopsies for men on active surveillance [20]. Increasing risk MPS2-AS results indicate a higher risk for GG≥2 and/or GG≥3 cancer. These patients can refer to their individual risk score to inform the decision to undergo prostate biopsy.

The aim of this study was to validate the analytical performance of MPS2-AS by measuring the reproducibility of MPS2-AS analyte detection and MPS2-AS risk scores across relevant within-laboratory parameters, as well as reagents and instruments.

## 2. Materials and Methods

### 2.1. Study Design and Pool Construction

Urine specimens were collected using the Colli-Pee™ urine collection device (DNA Genotek, Ottawa, ON, Canada) to standardize the collection of first-catch urine. Each collection captured 15 mL of first-catch urine into a urine preservation buffer, which stabilizes the specimen for ambient temperature transport without cold-chain requirements. After collection, specimens were packaged using the materials provided in the collection kit and shipped overnight to the Lynx Dx laboratory.

To evaluate the analytical precision and reproducibility across biologically relevant samples, urine specimens were pooled to provide sufficient volume per sample to evaluate MPS2-AS reproducibility. A total of 78 individual urine specimens were used to create 8 urine pools for testing, with 9–10 specimens included per pool. Urine samples were required to have a valid MPS2 result on original testing to ensure adequate prostate-derived RNA content. Pools were intentionally designed to exclusively include T2ERG-positive or T2ERG-negative samples to generate pools that spanned the range of MPS2-AS risk scores.

### 2.2. MPS2-AS RNA Extraction, Reverse Transcription, and Pre-Amplification PCR

MPS2-AS processing was performed as previously published [20]. Briefly, total RNA was isolated from 5 mL of urine. Reverse transcription was conducted immediately following extraction. Following reverse transcription, cDNA underwent multiplex target-specific pre-amplification PCR using standardized thermal cycling conditions. Pre-amplified PCR products were diluted 1:5 prior to quantitative PCR analysis.

### 2.3. Quantitative PCR and Plate Configuration

Quantitative PCR (qPCR) was performed using OpenArray™ chips on the QuantStudio™ 12K Flex Real-Time PCR System(Thermo Fisher Scientific, Waltham, MA, USA, QuantStudio 12K Flex software v1.4). Each OpenArray chip accommodates up to 48 samples per run. Cycle threshold (Crt) values for each MPS2-AS analyte were generated using instrument software with an automatic threshold.

### 2.4. MPS2-AS Algorithms

MPS2-AS predicts the risk of progression from GG1 to GG≥3 prostate cancer (MPS2-AS GG1-3 model) and GG1 to GG≥2 prostate cancer (MPS2-AS GG1-2 model) [14]. The MPS2-AS GG1-3 risk score is calculated from the qPCR results of KLK3, OR51E2, PCA3, PCAT14 and TRGV9, as well as the PSA density (PSAD). The MPS2-AS GG1-2 risk score is calculated from the qPCR results of KLK3, CAMKK2, PCA3, PCAT14, SCHLAP1, SPON2, TFF3, and TRGV9 and the TMPRSS-2:ERG fusion, as well as the PSA density (PSAD). Additionally, MPS2-AS thresholds were designed to stratify low-risk vs. increasing-risk patients and achieve sensitivity ≥90% and specificity ≥30% with an optimized NPV. An optimized MPS2-AS threshold of 11.5% was selected for both the GG1-2 and GG1-3 algorithms, as previously published [20].

### 2.5. Within-Laboratory Reproducibility Methods

To measure the MPS2-AS precision across days, runs and replicates, the MPS2-AS reproducibility was evaluated using eight urine pools over five testing days in an 8 × 5 × 2 × 2 design following the CLSI guidelines [25]. On each day, two independent RNA extractions were performed with each of the eight pools processed in duplicate, yielding four measurements per pool per day. Over five days, this design generated twenty measurements per pool and 160 total results to evaluate the MPS2-AS reproducibility.

### 2.6. Instrument-to-Instrument Reproducibility Methods

Instrument reproducibility was evaluated by analyzing pre-amplified material on three QuantStudio12K Flex systems in an 8 × 3 × 3 × 2 × 2 design following the CLSI guidelines [25]. Pre-amplified products from 8 urine pools were analyzed from three days of testing, which included two runs per day and 2 replicates per run. Each product was measured twelve times on three QuantStudio 12K Flex systems for a total of thirty-six results to assess variability attributable to qPCR instruments.

### 2.7. OpenArray Chip Lot Reproducibility Methods

OpenArray chip-to-chip reproducibility was evaluated using three independent OpenArray chip lots following the CLSI guidelines [25]. Pre-amplified products from 8 urine pools were analyzed from three days of testing, which included two runs per day and 2 replicates per run. Each product was measured twelve times on three OpenArray chip lots for a total of thirty-six results to assess the manufacturing variation across lots of OpenArray chips.

### 2.8. Statistical Analysis

Precision evaluation was performed using a hierarchical variance component analysis based on nested Analysis of Variance (ANOVA) to systematically partition the total variability into discrete components. No outliers were removed from the analysis. For the within-laboratory assessment, a two-way nested model was employed to estimate the variance contributions from between-day, between-run, and within-run sources. To evaluate the system-wide reproducibility across QuantStudio 12K Flex systems and OpenArray chip manufacturing lots, the framework was extended to a three-way nested ANOVA, accounting for the nesting of replicates within runs, runs within days, and days within either instruments or chip lots. Variance components were derived from the ANOVA framework to isolate independent variance estimates from each hierarchical level. The within-lab SD and reproducibility SD were calculated as the square root of the sum of these individual variance components. All data multi-level statistical modeling and variance component extractions were performed using Python (v3.9.20) utilizing the statsmodels library for the ANOVA framework [26].

## 3. Results

### 3.1. Within Laboratory Reproducibility

Within-laboratory reproducibility was analyzed in an 8 × 5 × 2 × 2 design that evaluated eight pooled urine samples over five days with two runs per day and two replicates per run. This resulted in a total of 20 sample-level results per sample pool. For each sample pool, ten MPS2-AS analytes (CAMKK2, KLK3, OR51E2, PCA3, PCAT14, SCHLAP1, SPON2, TFF3, TRGV9 and the T2ERG fusion) and two MPS2-AS scores (GG1-2 and GG1-3) were evaluated, totaling 1920 measurements (8 pools × 20 results/pool × 12 measurements/result) for the within-laboratory evaluation.

MPS2-AS analytes were evaluated for repeatability and between-run, between-day, and total within-lab precision, following the CLSI guidelines (Table 1). The MPS2-AS analyte measurements were highly reproducible across all within laboratory categories (repeatability, between-run, between-day and total). The MPS2-AS analyte measurements standard deviations and %CV ranged from 0.00 to 0.60 and 0.00% to 4.01% respectively, demonstrating the robustness of MPS2-AS analyte detection.

**Figure 1:**
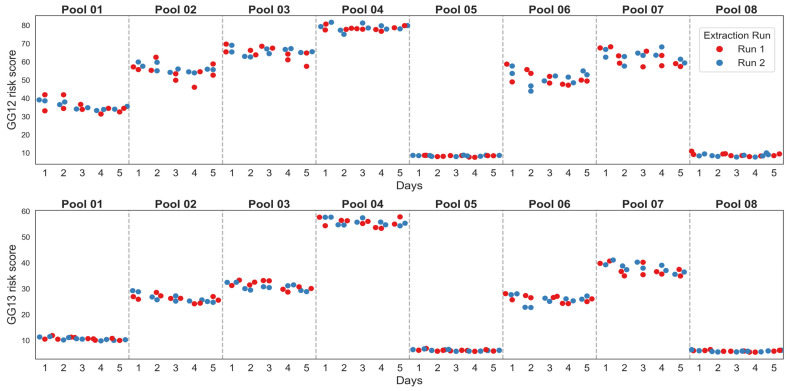
MPS2-AS GG1-2 and GG1-3 risk score within-lab reproducibility.

MPS2-AS analyte measurements were used to calculate MPS2-AS GG1-2 and GG1-3 risk scores and evaluate model reproducibility across the same parameters (Figure 1 and Table 2). MPS2-AS models similarly demonstrated strong reproducibility. The GG1-2 risk score standard deviation and %CV ranged from 0.36 to 3.95 and 2.05% to 9.98% respectively, while the GG1-3 risk score standard deviation and %CV ranged from 0.30 to 2.13 and 2.56% to 6.16% respectively (Table 2). Additionally, there were no apparent trends between runs or replicates performed across days suggesting no systematic bias in the results across these factors (Figure 1).

### 3.2. Instrument-to-Instrument Reproducibility

The instrument-to-instrument reproducibility was conducted in an 8 × 3 × 2 × 2 design that evaluated eight pooled urine samples over three days with two runs per day and two replicates per run. The pre-amplified product of each sample pool was measured twelve times on three QuantStudio 12K Flex systems for a total of 36 sample-level results to assess the variability attributable to qPCR instruments. For each sample pool, ten MPS2-AS analytes (CAMKK2, KLK3, OR51E2, PCA3, PCAT14, SCHLAP1, SPON2, TFF3, TRGV9 and the T2ERG fusion) and two MPS2-AS scores (GG1-2 and GG1-3) were evaluated, totaling 3456 measurements (8 pools × 36 results/pool × 12 measurements/result) for the instrument-to-instrument evaluation.

MPS2-AS analytes were evaluated for repeatability, between-run, between-day, within-lab, between-instrument, and reproducibility following the CLSI guidelines (Table 3). The MPS2-AS analyte measurements were highly reproducible across all within-laboratory categories (repeatability, between-run, between-day, within-lab, between-instrument, and reproducibility). The MPS2-AS analyte measurements standard deviations and %CV ranged from 0.00 to 0.68 and 0.00% to 4.57% respectively, demonstrating the robustness of MPS2-AS analyte detection between instruments.

**Figure 2:**
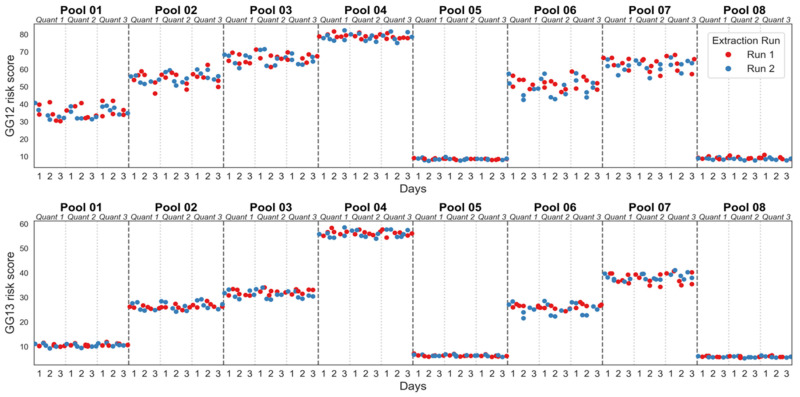
MPS2-AS GG1-2 and GG1-3 risk scores for instrument-to-instrument reproducibility.

The MPS2-AS analyte measurements were also used to calculate the MPS2-AS GG1-2 and GG1-3 risk scores and evaluate the model reproducibility across the same parameters (Figure 2 and Table 4). The MPS2-AS models were highly reproducible, where the GG1-2 model standard deviation and %CV ranged from 0.00 to 0.51 and 0.00% to 10.69% respectively, while the GG1-3 model standard deviation and %CV ranged from 0.00 to 2.01 and 0.00% to 7.20% respectively (Table 4). There were no apparent trends between the three instruments suggesting no systemic bias in the results across these factors (Figure 2).

### 3.3. OpenArray Chip Lot Reproducibility

The OpenArray chip lot reproducibility was conducted in an 8 × 3 × 2 × 2 design that evaluated eight pool urine samples over three days, with two runs per day and two replicates per run. The pre-amplified product was run on three lots of OpenArray chips on the same QuantStudio 12K Flex System, generating a total of 36 sample-level results for each sample pool to assess the lot-to-lot variation. For each sample pool, ten MPS2-AS analytes (CAMKK2, KLK3, OR51E2, PCA3, PCAT14, SCHLAP1, SPON2, TFF3, TRGV9 and the T2ERG fusion) and two MPS2-AS scores (GG1-2 and GG1-3) were evaluated, totaling 3456 measurements (8 pools × 36 results/pool × 12 measurements/result) for the chip lot evaluation.

MPS2-AS analytes were evaluated for repeatability, between-run, between-day, within-lab, between-lot, and reproducibility following the CLSI guidelines (Table 5). The MPS2-AS analyte measurements were highly reproducible between the three chip lots (repeatability, between-run, between-day, within-lab, between-lot, and reproducibility). The MPS2-AS analyte measurements standard deviations and %CV ranged from 0.00 to 0.71 and 0.00% to 4.10% respectively, demonstrating the robustness in MPS2-AS analyte detection across chip lots.

**Figure 3:**
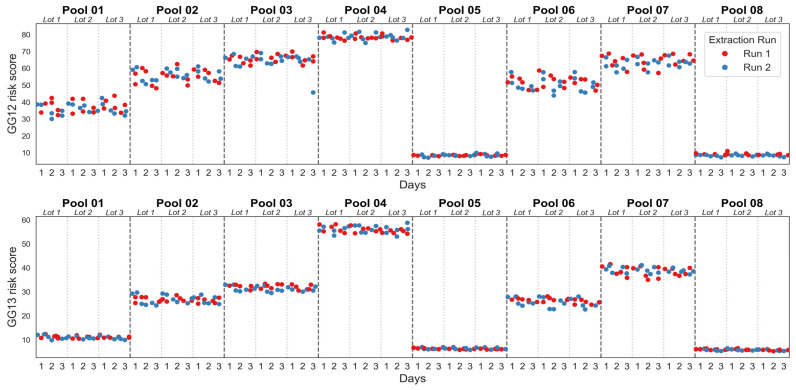
MPS2-AS GG1-2 and GG1-3 risk scores for reproducibility across reagent lots.

The MPS2-AS analyte measurements were also used to calculate the MPS2-AS GG1-2 and GG1-3 risk scores and evaluate the model reproducibility across the same parameters (Figure 3 and Table 6). The MPS2-AS models were highly reproducible between chip lots, where the GG1-2 model standard deviation and %CV ranged from 0.00 to 4.17 and 0.00% to 9.83% respectively, while the GG1-3 model standard deviation and %CV ranged from 0.00 to 1.90 and 0.00% to 6.29% respectively (Table 6). There were no apparent trends between the three chip lots, suggesting no systemic bias in the results across these factors (Figure 3).

## 4. Discussion

There is a significant unmet need for non-invasive diagnostic tools to guide biopsy decisions for men on active surveillance for prostate cancer [20,27]. MPS2-AS has been designed to fill this gap by predicting the risk for GG≥2 and GG≥3 prostate cancers in men on active surveillance with GG1 cancer. MPS2-AS has been clinically validated to rule out GG≥2 and GG≥3 prostate cancers at NPVs of 92% and 99% respectively, providing an effective tool to reasonably defer biopsies for men on active surveillance [20].

This analytical validation builds upon a previous MPS2 analytical validation, which defined the limits of detection, linearity, and RT-PCR reproducibility of MPS2 analyte amplification [23]. Since MPS2 and MPS2-AS laboratory protocols have the same methods, reagents and equipment, with MPS2-AS measuring a subset of the MPS2 panel, these previous MPS2 results similarly apply to MPS2-AS.

The aim of this study was to analytically validate the accuracy and robustness of MPS2-AS to conventional variation that occurs in a clinical laboratory. The study was designed following CLSI principles to evaluate MPS2-AS reproducibility at a single laboratory by examining within-run (repeatability), between-run and between-day variations in an 8 × 5 × 2 × 2 design. MPS2-AS analyte amplification was shown to be highly reproducible across these metrics, with %CV ≤ 4.01%. This outperforms the within-laboratory analytical precision of another non-invasive prostate cancer screening test, the IsoPSA Assay, which has %CV ≤ 9.1% [28].

The MPS2-AS RT-PCR is conducted on the OpenArray system, which performs up to 3072 RT-PCR reactions in 33 nL reactions across 48 samples per run. RT-PCR reactions are performed on custom OpenArray chips designed to measure the MPS2-AS biomarker panels on the QuantStudio12K System. Since this critical step in the MPS2-AS protocol requires custom reagents and highly specialized and sensitive equipment, it was identified as the highest risk step for MPS2-AS reproducibility. When MPS2-AS reproducibility was evaluated across three lots of OpenArray chips and three QuantStudio12K Systems, MPS2-AS showed strong reproducibility with %CV ≤ 4.10% and %CV ≤ 4.57% across reagents and equipment respectively, confirming assay robustness. MPS2-AS analyte-level reproducibility is similar to the lot-to-lot analytical precision of the IsoPSA Assay, which has %CV ≤ 4.7% [28].

MPS2-AS risk stratifies men for GG≥2 and GG≥3 cancer using the validated threshold of 11.5% [20]. Importantly, this validation examined MPS2-AS GG1-2 and GG1-3 score reproducibility at values near this threshold, confirming the robustness of this assay near the clinical threshold.

This robust MPS2-AS analyte precision translated into highly reproducible MPS2-AS GG1-2 and GG1-3 risk scores. Across within-laboratory, between-reagent-lot and between-instrument sources, the MPS2-AS GG1-2 and GG1-3 model scores were highly reproducible with %CV ≤ 10.69% and %CV ≤ 7.20% respectively. The MPS2-AS GG1-3 utilizes a five-biomarker panel whereas MPS2-AS GG1-2 is a nine-biomarker gene panel. The additional biomarkers used in the GG1-2 model drives a slight increase in GG1-2 risk score %CV compared with GG1-3 risk scores. Nevertheless, both GG1-2 and GG1-3 risk scores outperformed the IsoPSA Assay score reproducibility, which had %CV values up to 16.8% [28]. Another limitation is the use of pooled specimen for analytical validation. While pooling was necessary to generate the required replicates for comprehensive reproducibility studies, this may obscure some of the patient-level variability associated with prostate cancer that would occur with routine MPS2-AS testing.

## 5. Conclusions

This analytical validation confirms the reproducibility and precision of MPS2-AS for quantifying the 10 target MPS2-AS biomarkers and calculating GG1-2 and GG1-3 model scores across conventional sources of within-laboratory variation, as well as high risk reagents and equipment. The precision and reliability of MPS2-AS results demonstrated in this study, coupled with the clinical performance of MPS2-AS to rule out progression to GG≥2 and GG≥3 prostate cancer further supports the use of MPS2-AS to inform biopsy decision for men with GG1 prostate cancer on active surveillance.

## Figures and Tables

**Table 1:** MPS2-AS analyte results for within-lab reproducibility and variance contributions from between-day, between-run and between-replicate (repeatability) sources.

				Repeatability		Between-Run		Between-Day		Within-Lab (Total)	
	Pool ID	Mean	N	SD	%CV	SD	%CV	SD	%CV	SD	%CV
CAMKK2	Pool 05	18.35	20	0.18	0.97	0.15	0.83	0.13	0.73	0.27	1.47
CAMKK2	Pool 01	18.17	20	0.16	0.88	0.00	0.00	0.12	0.66	0.20	1.10
CAMKK2	Pool 02	17.17	20	0.15	0.90	0.00	0.00	0.15	0.87	0.21	1.25
CAMKK2	Pool 04	17.81	20	0.27	1.53	0.09	0.52	0.08	0.47	0.30	1.69
CAMKK2	Pool 06	19.71	20	0.14	0.70	0.11	0.57	0.09	0.46	0.20	1.01
CAMKK2	Pool 08	19.88	20	0.19	0.96	0.13	0.66	0.17	0.85	0.29	1.44
CAMKK2	Pool 03	16.87	20	0.21	1.24	0.06	0.33	0.13	0.79	0.25	1.50
CAMKK2	Pool 07	19.35	20	0.21	1.08	0.00	0.00	0.09	0.48	0.23	1.19
KLK3	Pool 05	14.73	20	0.13	0.89	0.18	1.23	0.00	0.00	0.22	1.52
KLK3	Pool 08	15.70	20	0.10	0.62	0.13	0.83	0.07	0.47	0.18	1.14
KLK3	Pool 04	14.05	20	0.37	2.64	0.26	1.83	0.00	0.00	0.45	3.21
KLK3	Pool 01	14.16	20	0.15	1.08	0.00	0.00	0.07	0.47	0.17	1.18
KLK3	Pool 07	16.07	20	0.27	1.65	0.00	0.00	0.00	0.00	0.27	1.65
KLK3	Pool 06	15.39	20	0.22	1.45	0.24	1.58	0.00	0.00	0.33	2.14
KLK3	Pool 02	13.08	20	0.13	1.01	0.18	1.37	0.00	0.00	0.22	1.70
KLK3	Pool 03	12.61	20	0.16	1.25	0.21	1.64	0.00	0.00	0.26	2.06
OR51E2	Pool 04	18.23	20	0.33	1.79	0.15	0.85	0.00	0.00	0.36	1.98
OR51E2	Pool 01	18.62	20	0.11	0.61	0.14	0.76	0.06	0.30	0.19	1.02
OR51E2	Pool 05	19.03	20	0.18	0.93	0.11	0.60	0.09	0.47	0.23	1.20
OR51E2	Pool 07	20.91	20	0.15	0.73	0.20	0.97	0.00	0.00	0.25	1.21
OR51E2	Pool 08	20.90	20	0.17	0.81	0.20	0.93	0.17	0.83	0.31	1.49
OR51E2	Pool 02	18.03	20	0.15	0.85	0.00	0.00	0.12	0.66	0.19	1.08
OR51E2	Pool 06	21.21	20	0.27	1.27	0.26	1.25	0.00	0.00	0.38	1.78
OR51E2	Pool 03	17.50	20	0.17	0.97	0.00	0.00	0.17	0.98	0.24	1.38
PCA3	Pool 05	17.76	20	0.10	0.54	0.14	0.81	0.00	0.00	0.17	0.97
PCA3	Pool 07	19.76	20	0.13	0.67	0.08	0.40	0.04	0.18	0.16	0.80
PCA3	Pool 06	19.30	20	0.14	0.74	0.15	0.79	0.00	0.00	0.21	1.08
PCA3	Pool 08	20.30	20	0.17	0.83	0.13	0.66	0.00	0.00	0.22	1.06
PCA3	Pool 04	15.72	20	0.31	1.95	0.10	0.63	0.06	0.40	0.33	2.09
PCA3	Pool 01	17.98	20	0.07	0.40	0.08	0.44	0.05	0.26	0.12	0.65
PCA3	Pool 03	15.98	20	0.17	1.04	0.13	0.83	0.06	0.35	0.22	1.38
PCA3	Pool 02	16.39	20	0.12	0.74	0.07	0.45	0.07	0.43	0.16	0.97
PCAT14	Pool 06	21.56	20	0.20	0.94	0.11	0.52	0.00	0.00	0.23	1.08
PCAT14	Pool 02	20.05	20	0.14	0.70	0.10	0.49	0.03	0.17	0.17	0.87
PCAT14	Pool 08	23.40	20	0.25	1.06	0.00	0.00	0.26	1.09	0.36	1.52
PCAT14	Pool 01	20.82	20	0.15	0.70	0.04	0.18	0.06	0.29	0.16	0.78
PCAT14	Pool 07	22.72	20	0.20	0.88	0.00	0.00	0.05	0.22	0.21	0.91
PCAT14	Pool 03	19.66	20	0.14	0.70	0.15	0.74	0.08	0.43	0.22	1.11
PCAT14	Pool 05	23.59	20	0.32	1.37	0.24	1.03	0.00	0.00	0.40	1.71
PCAT14	Pool 04	18.11	20	0.30	1.65	0.15	0.80	0.00	0.00	0.33	1.83
SCHLAP1	Pool 02	21.49	20	0.15	0.68	0.00	0.00	0.11	0.49	0.18	0.84
SCHLAP1	Pool 05	23.06	20	0.29	1.27	0.00	0.00	0.24	1.06	0.38	1.65
SCHLAP1	Pool 03	20.60	20	0.21	1.04	0.06	0.31	0.19	0.90	0.29	1.41
SCHLAP1	Pool 07	24.18	20	0.40	1.64	0.00	0.00	0.29	1.20	0.49	2.03
SCHLAP1	Pool 04	21.16	20	0.46	2.17	0.00	0.00	0.21	1.01	0.51	2.39
SCHLAP1	Pool 08	23.24	20	0.30	1.28	0.05	0.21	0.39	1.68	0.49	2.13
SCHLAP1	Pool 06	22.79	20	0.36	1.58	0.14	0.61	0.21	0.92	0.44	1.93
SCHLAP1	Pool 01	21.47	20	0.22	1.01	0.00	0.00	0.14	0.65	0.26	1.20
SPON2	Pool 02	18.78	20	0.15	0.82	0.05	0.26	0.02	0.12	0.16	0.87
SPON2	Pool 04	19.29	20	0.56	2.92	0.22	1.12	0.00	0.00	0.60	3.12
SPON2	Pool 06	20.80	20	0.33	1.60	0.28	1.34	0.00	0.00	0.43	2.09
SPON2	Pool 03	18.69	20	0.26	1.38	0.15	0.82	0.00	0.00	0.30	1.61
SPON2	Pool 01	19.22	20	0.13	0.66	0.14	0.75	0.02	0.11	0.19	1.00
SPON2	Pool 08	21.74	20	0.18	0.82	0.14	0.63	0.06	0.25	0.23	1.06
SPON2	Pool 05	19.10	20	0.18	0.96	0.39	2.02	0.00	0.00	0.43	2.24
SPON2	Pool 07	21.10	20	0.28	1.34	0.00	0.00	0.00	0.00	0.28	1.34
T2ERG	Pool 06	28.00	20	0.00	0.00	0.00	0.00	0.00	0.00	0.00	0.00
T2ERG	Pool 08	27.91	20	0.39	1.39	0.00	0.00	0.00	0.00	0.39	1.39
T2ERG	Pool 02	23.45	20	0.50	2.14	0.00	0.00	0.28	1.18	0.57	2.45
T2ERG	Pool 03	23.77	20	0.47	1.99	0.31	1.28	0.10	0.42	0.57	2.41
T2ERG	Pool 05	28.00	20	0.00	0.00	0.00	0.00	0.00	0.00	0.00	0.00
T2ERG	Pool 07	28.00	20	0.00	0.00	0.00	0.00	0.00	0.00	0.00	0.00
T2ERG	Pool 04	28.00	20	0.00	0.00	0.00	0.00	0.00	0.00	0.00	0.00
T2ERG	Pool 01	23.30	20	0.28	1.19	0.07	0.29	0.17	0.73	0.33	1.42
TFF3	Pool 01	18.79	20	0.16	0.86	0.00	0.00	0.00	0.00	0.16	0.86
TFF3	Pool 07	20.57	20	0.23	1.13	0.00	0.00	0.12	0.58	0.26	1.27
TFF3	Pool 06	21.38	20	0.30	1.40	0.21	0.98	0.00	0.00	0.36	1.71
TFF3	Pool 02	18.21	20	0.17	0.93	0.00	0.00	0.13	0.72	0.21	1.18
TFF3	Pool 03	17.14	20	0.21	1.23	0.07	0.41	0.10	0.56	0.24	1.41
TFF3	Pool 05	19.17	20	0.20	1.02	0.17	0.86	0.00	0.00	0.26	1.34
TFF3	Pool 04	18.39	20	0.44	2.42	0.00	0.00	0.17	0.93	0.48	2.59
TFF3	Pool 08	21.25	20	0.19	0.88	0.24	1.13	0.05	0.22	0.31	1.45
TRGV9	Pool 04	14.55	20	0.52	3.54	0.27	1.88	0.00	0.00	0.58	4.01
TRGV9	Pool 06	16.42	20	0.31	1.87	0.23	1.40	0.00	0.00	0.38	2.33
TRGV9	Pool 05	16.07	20	0.18	1.14	0.29	1.78	0.00	0.00	0.34	2.11
TRGV9	Pool 03	13.60	20	0.22	1.60	0.20	1.45	0.00	0.00	0.29	2.16
TRGV9	Pool 02	14.15	20	0.19	1.35	0.22	1.57	0.00	0.00	0.29	2.07
TRGV9	Pool 01	14.64	20	0.11	0.73	0.05	0.37	0.05	0.36	0.13	0.89
TRGV9	Pool 07	17.36	20	0.34	1.99	0.00	0.00	0.00	0.00	0.34	1.99
TRGV9	Pool 08	16.50	20	0.13	0.78	0.19	1.16	0.16	1.00	0.28	1.72

**Table 2:** MPS2-AS risk score within-lab reproducibility and variance contributions from between-day, between-run and between-replicate (repeatability) sources.

				Repeatability		Between-Run		Between-Day		Within-Lab (Total)		
MPS2-AS Model	Pool ID	Mean	N	SD	%CV	SD	%CV	SD	%CV	SD	%CV	Psad
GG1-2	Pool 01	35.60	20	2.84	7.97	0.00	0.00	2.14	6.01	3.55	9.98	0.10
GG1-2	Pool 02	55.42	20	3.23	5.82	0.00	0.00	2.21	3.99	3.91	7.06	0.30
GG1-2	Pool 03	65.25	20	2.34	3.58	1.37	2.11	1.01	1.54	2.89	4.43	0.34
GG1-2	Pool 04	78.60	20	1.37	1.75	0.51	0.65	0.68	0.86	1.62	2.05	0.40
GG1-2	Pool 05	8.36	20	0.22	2.62	0.17	2.03	0.23	2.76	0.36	4.31	0.02
GG1-2	Pool 06	51.15	20	2.83	5.53	2.76	5.40	0.00	0.00	3.95	7.73	0.32
GG1-2	Pool 07	62.48	20	3.13	5.01	0.39	0.63	2.13	3.41	3.81	6.10	0.40
GG1-2	Pool 08	8.81	20	0.63	7.15	0.33	3.69	0.42	4.83	0.83	9.38	0.04
GG1-3	Pool 01	10.63	20	0.50	4.70	0.00	0.00	0.42	3.98	0.65	6.16	0.10
GG1-3	Pool 02	26.28	20	0.73	2.77	1.01	3.85	0.81	3.09	1.49	5.66	0.30
GG1-3	Pool 03	31.01	20	0.62	2.02	1.24	4.01	0.55	1.78	1.50	4.83	0.34
GG1-3	Pool 04	55.72	20	1.11	1.99	0.77	1.37	0.46	0.82	1.42	2.56	0.40
GG1-3	Pool 05	6.17	20	0.29	4.62	0.00	0.00	0.23	3.75	0.37	5.95	0.02
GG1-3	Pool 06	25.89	20	0.76	2.93	1.40	5.42	0.00	0.00	1.59	6.16	0.32
GG1-3	Pool 07	37.75	20	1.58	4.17	0.00	0.00	1.44	3.81	2.13	5.65	0.40
GG1-3	Pool 08	5.89	20	0.22	3.78	0.09	1.49	0.18	3.03	0.30	5.07	0.04

**Table 3:** MPS2-AS analyte results for between-instrument, within-lab reproducibility and variance contributions from between-day, between-run and between-replicate (repeatability) and overall reproducibility sources.

				Repeatability		Between-Run		Between-Day		Within-Lab		Between-Instrument		Reproducibility	
	Pool ID	Mean	N	SD	%CV	SD	%CV	SD	%CV	SD	%CV	SD	%CV	SD	%CV
CAMKK2	Pool 05	18.20	36	0.18	1.00	0.18	0.99	0.07	0.37	0.27	1.46	0.00	0.00	0.27	1.46
CAMKK2	Pool 01	18.12	36	0.14	0.77	0.00	0.00	0.19	1.06	0.24	1.32	0.00	0.00	0.24	1.32
CAMKK2	Pool 02	17.14	36	0.19	1.08	0.14	0.83	0.18	1.03	0.29	1.71	0.00	0.00	0.29	1.71
CAMKK2	Pool 04	17.77	36	0.34	1.90	0.13	0.71	0.16	0.89	0.39	2.22	0.00	0.00	0.39	2.22
CAMKK2	Pool 06	19.59	36	0.12	0.63	0.11	0.56	0.09	0.47	0.19	0.96	0.00	0.00	0.19	0.96
CAMKK2	Pool 08	19.75	36	0.08	0.41	0.17	0.88	0.00	0.00	0.19	0.97	0.00	0.00	0.19	0.97
CAMKK2	Pool 03	16.77	36	0.17	1.02	0.11	0.68	0.13	0.76	0.24	1.44	0.00	0.00	0.24	1.44
CAMKK2	Pool 07	19.25	36	0.22	1.16	0.00	0.00	0.10	0.50	0.24	1.27	0.06	0.30	0.25	1.30
KLK3	Pool 05	14.68	36	0.10	0.69	0.20	1.36	0.00	0.00	0.22	1.53	0.08	0.52	0.24	1.62
KLK3	Pool 08	15.61	36	0.11	0.72	0.10	0.63	0.04	0.23	0.15	0.98	0.07	0.46	0.17	1.09
KLK3	Pool 04	14.00	36	0.40	2.89	0.28	2.01	0.00	0.00	0.49	3.52	0.00	0.00	0.49	3.52
KLK3	Pool 01	14.09	36	0.19	1.32	0.00	0.00	0.00	0.00	0.19	1.32	0.10	0.74	0.21	1.51
KLK3	Pool 07	16.00	36	0.27	1.68	0.00	0.00	0.02	0.11	0.27	1.68	0.10	0.61	0.29	1.79
KLK3	Pool 06	15.27	36	0.20	1.32	0.28	1.85	0.00	0.00	0.35	2.27	0.09	0.59	0.36	2.34
KLK3	Pool 02	13.03	36	0.18	1.37	0.24	1.83	0.00	0.00	0.30	2.29	0.13	0.97	0.32	2.49
KLK3	Pool 03	12.53	36	0.12	0.94	0.24	1.95	0.00	0.00	0.27	2.17	0.07	0.56	0.28	2.24
OR51E2	Pool 04	18.12	36	0.33	1.82	0.14	0.80	0.12	0.68	0.38	2.10	0.00	0.00	0.38	2.10
OR51E2	Pool 01	18.53	36	0.15	0.80	0.11	0.58	0.00	0.00	0.18	0.99	0.04	0.20	0.19	1.01
OR51E2	Pool 05	18.88	36	0.16	0.83	0.11	0.59	0.00	0.00	0.19	1.02	0.05	0.29	0.20	1.06
OR51E2	Pool 07	20.82	36	0.17	0.79	0.21	0.99	0.00	0.00	0.26	1.27	0.00	0.00	0.26	1.27
OR51E2	Pool 08	20.80	36	0.16	0.78	0.23	1.09	0.00	0.00	0.28	1.34	0.00	0.00	0.28	1.34
OR51E2	Pool 02	17.93	36	0.17	0.96	0.00	0.00	0.07	0.42	0.19	1.05	0.07	0.42	0.20	1.13
OR51E2	Pool 06	21.09	36	0.21	1.02	0.27	1.30	0.00	0.00	0.35	1.65	0.04	0.19	0.35	1.66
OR51E2	Pool 03	17.33	36	0.17	0.97	0.00	0.00	0.14	0.82	0.22	1.27	0.05	0.27	0.23	1.30
PCA3	Pool 05	17.67	36	0.14	0.81	0.17	0.94	0.00	0.00	0.22	1.24	0.09	0.52	0.24	1.35
PCA3	Pool 07	19.68	36	0.19	0.96	0.14	0.69	0.00	0.00	0.23	1.19	0.10	0.52	0.25	1.30
PCA3	Pool 06	19.19	36	0.16	0.86	0.15	0.78	0.00	0.00	0.22	1.16	0.11	0.58	0.25	1.30
PCA3	Pool 08	20.18	36	0.14	0.69	0.10	0.51	0.00	0.00	0.17	0.86	0.09	0.45	0.20	0.97
PCA3	Pool 04	15.65	36	0.40	2.58	0.08	0.50	0.18	1.17	0.45	2.88	0.00	0.00	0.45	2.88
PCA3	Pool 01	17.92	36	0.09	0.51	0.04	0.24	0.11	0.61	0.15	0.83	0.06	0.35	0.16	0.90
PCA3	Pool 03	15.84	36	0.18	1.17	0.17	1.05	0.04	0.28	0.25	1.60	0.05	0.33	0.26	1.63
PCA3	Pool 02	16.32	36	0.14	0.88	0.15	0.93	0.07	0.40	0.22	1.34	0.11	0.67	0.24	1.50
PCAT14	Pool 06	21.50	36	0.19	0.91	0.19	0.89	0.00	0.00	0.27	1.27	0.06	0.29	0.28	1.31
PCAT14	Pool 02	20.05	36	0.14	0.71	0.19	0.96	0.00	0.00	0.24	1.19	0.04	0.18	0.24	1.21
PCAT14	Pool 08	23.41	36	0.26	1.11	0.00	0.00	0.19	0.82	0.32	1.38	0.00	0.00	0.32	1.38
PCAT14	Pool 01	20.76	36	0.17	0.83	0.00	0.00	0.08	0.41	0.19	0.92	0.02	0.07	0.19	0.92
PCAT14	Pool 07	22.66	36	0.31	1.36	0.09	0.40	0.08	0.33	0.33	1.45	0.00	0.00	0.33	1.45
PCAT14	Pool 03	19.54	36	0.16	0.80	0.16	0.80	0.22	1.15	0.32	1.61	0.00	0.00	0.32	1.61
PCAT14	Pool 05	23.47	36	0.34	1.47	0.22	0.96	0.20	0.86	0.46	1.95	0.00	0.00	0.46	1.95
PCAT14	Pool 04	18.04	36	0.31	1.69	0.19	1.06	0.16	0.90	0.40	2.19	0.00	0.00	0.40	2.19
SCHLAP1	Pool 02	21.50	36	0.15	0.71	0.05	0.24	0.23	1.08	0.28	1.32	0.00	0.00	0.28	1.32
SCHLAP1	Pool 05	22.84	36	0.26	1.15	0.00	0.00	0.24	1.04	0.35	1.55	0.00	0.00	0.35	1.55
SCHLAP1	Pool 03	20.43	36	0.13	0.62	0.08	0.39	0.23	1.13	0.28	1.35	0.00	0.00	0.28	1.35
SCHLAP1	Pool 07	24.01	36	0.40	1.67	0.00	0.00	0.36	1.51	0.54	2.26	0.00	0.00	0.54	2.26
SCHLAP1	Pool 04	21.11	36	0.46	2.19	0.00	0.00	0.33	1.54	0.57	2.68	0.00	0.00	0.57	2.68
SCHLAP1	Pool 08	22.97	36	0.24	1.02	0.26	1.11	0.00	0.00	0.35	1.51	0.00	0.00	0.35	1.51
SCHLAP1	Pool 06	22.61	36	0.38	1.70	0.20	0.88	0.00	0.00	0.43	1.92	0.00	0.00	0.43	1.92
SCHLAP1	Pool 01	21.40	36	0.22	1.01	0.00	0.00	0.21	0.97	0.30	1.40	0.00	0.00	0.30	1.40
SPON2	Pool 02	18.77	36	0.19	1.01	0.10	0.55	0.10	0.54	0.24	1.27	0.00	0.00	0.24	1.27
SPON2	Pool 04	19.19	36	0.51	2.67	0.42	2.18	0.18	0.92	0.68	3.57	0.00	0.00	0.68	3.57
SPON2	Pool 06	20.73	36	0.31	1.52	0.21	1.01	0.08	0.41	0.39	1.87	0.00	0.00	0.39	1.87
SPON2	Pool 03	18.54	36	0.24	1.32	0.10	0.56	0.03	0.17	0.27	1.44	0.09	0.47	0.28	1.52
SPON2	Pool 01	19.14	36	0.13	0.70	0.07	0.38	0.16	0.85	0.22	1.17	0.00	0.00	0.22	1.17
SPON2	Pool 08	21.67	36	0.21	0.98	0.19	0.88	0.00	0.00	0.29	1.32	0.00	0.00	0.29	1.32
SPON2	Pool 05	18.97	36	0.16	0.84	0.40	2.11	0.00	0.00	0.43	2.27	0.08	0.44	0.44	2.32
SPON2	Pool 07	21.02	36	0.33	1.56	0.00	0.00	0.11	0.52	0.35	1.65	0.00	0.00	0.35	1.65
T2ERG	Pool 06	28.00	36	0.00	0.00	0.00	0.00	0.00	0.00	0.00	0.00	0.00	0.00	0.00	0.00
T2ERG	Pool 08	27.92	36	0.33	1.18	0.00	0.00	0.00	0.00	0.33	1.18	0.00	0.00	0.33	1.18
T2ERG	Pool 02	23.47	36	0.37	1.59	0.08	0.36	0.26	1.10	0.46	1.96	0.00	0.00	0.46	1.96
T2ERG	Pool 03	23.70	36	0.49	2.07	0.00	0.00	0.44	1.84	0.66	2.77	0.00	0.00	0.66	2.77
T2ERG	Pool 05	28.00	36	0.00	0.00	0.00	0.00	0.00	0.00	0.00	0.00	0.00	0.00	0.00	0.00
T2ERG	Pool 07	28.00	36	0.00	0.00	0.00	0.00	0.00	0.00	0.00	0.00	0.00	0.00	0.00	0.00
T2ERG	Pool 04	28.00	36	0.00	0.00	0.00	0.00	0.00	0.00	0.00	0.00	0.00	0.00	0.00	0.00
T2ERG	Pool 01	23.26	36	0.31	1.35	0.00	0.00	0.31	1.34	0.44	1.90	0.00	0.00	0.44	1.90
TFF3	Pool 01	18.75	36	0.16	0.86	0.00	0.00	0.05	0.27	0.17	0.90	0.07	0.35	0.18	0.97
TFF3	Pool 07	20.53	36	0.25	1.23	0.00	0.00	0.16	0.77	0.30	1.45	0.00	0.00	0.30	1.45
TFF3	Pool 06	21.27	36	0.26	1.22	0.25	1.16	0.00	0.00	0.36	1.68	0.03	0.15	0.36	1.69
TFF3	Pool 02	18.16	36	0.16	0.87	0.01	0.08	0.13	0.70	0.20	1.12	0.06	0.30	0.21	1.16
TFF3	Pool 03	17.03	36	0.17	1.01	0.11	0.66	0.12	0.68	0.24	1.38	0.04	0.23	0.24	1.40
TFF3	Pool 05	19.08	36	0.14	0.75	0.16	0.86	0.00	0.00	0.22	1.14	0.06	0.29	0.22	1.18
TFF3	Pool 04	18.33	36	0.50	2.72	0.00	0.00	0.27	1.47	0.57	3.09	0.00	0.00	0.57	3.09
TFF3	Pool 08	21.12	36	0.13	0.63	0.28	1.32	0.00	0.00	0.31	1.46	0.00	0.00	0.31	1.46
TRGV9	Pool 04	14.49	36	0.57	3.92	0.33	2.28	0.08	0.53	0.66	4.57	0.00	0.00	0.66	4.57
TRGV9	Pool 06	16.29	36	0.26	1.62	0.34	2.09	0.00	0.00	0.43	2.64	0.06	0.39	0.44	2.67
TRGV9	Pool 05	15.94	36	0.17	1.04	0.29	1.83	0.00	0.00	0.34	2.10	0.09	0.58	0.35	2.18
TRGV9	Pool 03	13.44	36	0.19	1.42	0.17	1.26	0.11	0.80	0.28	2.06	0.00	0.00	0.28	2.06
TRGV9	Pool 02	14.09	36	0.21	1.52	0.25	1.76	0.00	0.00	0.33	2.33	0.12	0.83	0.35	2.47
TRGV9	Pool 01	14.60	36	0.15	1.06	0.00	0.00	0.04	0.28	0.16	1.10	0.05	0.37	0.17	1.16
TRGV9	Pool 07	17.27	36	0.38	2.22	0.00	0.00	0.15	0.89	0.41	2.39	0.00	0.00	0.41	2.39
TRGV9	Pool 08	16.36	36	0.16	0.99	0.14	0.85	0.11	0.65	0.24	1.46	0.03	0.17	0.24	1.47

**Table 4:** MPS2-AS risk scores for between-instrument, within-lab reproducibility and variance contributions from between-day, between-run and between-replicate (repeatability) sources.

				Repeatability		Between-Run		Between-Day		Within-Lab		Between-Lot		Reproducibility		
	Pool ID	Mean	N	SD	%CV	SD	%CV	SD	%CV	SD	%CV	SD	%CV	SD	%CV	Psad
GG1-2	Pool 01	35.42	36	3.07	8.67	0.00	0.00	2.21	6.25	3.79	10.69	0.00	0.00	3.79	10.69	0.10
GG1-2	Pool 02	55.00	36	2.22	4.04	2.10	3.82	1.79	3.26	3.54	6.45	0.00	0.00	3.54	6.45	0.30
GG1-2	Pool 03	66.14	36	2.20	3.32	0.65	0.98	2.16	3.27	3.15	4.76	0.00	0.00	3.15	4.76	0.34
GG1-2	Pool 04	78.74	36	1.66	2.10	0.08	0.10	0.76	0.96	1.82	2.31	0.00	0.00	1.82	2.31	0.40
GG1-2	Pool 05	8.51	36	0.30	3.52	0.18	2.12	0.38	4.42	0.51	6.04	0.00	0.00	0.51	6.04	0.02
GG1-2	Pool 06	50.75	36	2.80	5.51	3.59	7.06	0.00	0.00	4.55	8.96	0.00	0.00	4.55	8.96	0.32
GG1-2	Pool 07	62.50	36	3.16	5.06	0.00	0.00	2.36	3.77	3.94	6.31	0.00	0.00	3.94	6.31	0.40
GG1-2	Pool 08	8.93	36	0.60	6.68	0.46	5.13	0.04	0.48	0.75	8.44	0.00	0.00	0.75	8.44	0.04
GG1-3	Pool 01	10.61	36	0.49	4.60	0.00	0.00	0.38	3.57	0.62	5.82	0.04	0.39	0.62	5.84	0.10
GG1-3	Pool 02	26.35	36	0.73	2.77	1.02	3.86	0.20	0.76	1.27	4.81	0.30	1.15	1.30	4.95	0.30
GG1-3	Pool 03	31.70	36	0.88	2.78	1.03	3.26	0.58	1.84	1.48	4.66	0.00	0.00	1.48	4.66	0.34
GG1-3	Pool 04	56.12	36	1.08	1.93	0.72	1.29	0.00	0.00	1.30	2.32	0.00	0.00	1.30	2.32	0.40
GG1-3	Pool 05	6.38	36	0.26	4.14	0.00	0.00	0.30	4.77	0.40	6.32	0.00	0.00	0.40	6.32	0.02
GG1-3	Pool 06	25.78	36	0.80	3.12	1.62	6.29	0.42	1.62	1.86	7.20	0.00	0.00	1.86	7.20	0.32
GG1-3	Pool 07	37.99	36	1.54	4.05	0.00	0.00	1.30	3.42	2.01	5.30	0.00	0.00	2.01	5.30	0.40
GG1-3	Pool 08	5.91	36	0.23	3.90	0.05	0.92	0.17	2.95	0.29	4.98	0.00	0.00	0.29	4.98	0.04

**Table 5:** MPS2-AS analyte results for between-reagent-lot, within-lab reproducibility and variance contributions from between-day, between-run and between-replicate (repeatability) and overall reproducibility sources.

				Repeatability		Between-Run		Between-Day		Within-Lab		Between-Lot		Reproducibility	
	Pool ID	Mean	N	SD	%CV	SD	%CV	SD	%CV	SD	%CV	SD	%CV	SD	%CV
CAMKK2	Pool 05	18.27	36	0.14	0.76	0.17	0.94	0.11	0.59	0.25	1.35	0.00	0.00	0.25	1.35
CAMKK2	Pool 01	18.22	36	0.15	0.80	0.05	0.25	0.22	1.23	0.27	1.49	0.00	0.00	0.27	1.49
CAMKK2	Pool 02	17.24	36	0.15	0.85	0.14	0.83	0.19	1.10	0.28	1.62	0.00	0.00	0.28	1.62
CAMKK2	Pool 04	17.83	36	0.33	1.83	0.01	0.08	0.15	0.87	0.36	2.03	0.00	0.00	0.36	2.03
CAMKK2	Pool 06	19.67	36	0.10	0.50	0.07	0.37	0.12	0.59	0.17	0.86	0.00	0.00	0.17	0.86
CAMKK2	Pool 08	19.79	36	0.12	0.60	0.07	0.35	0.08	0.38	0.16	0.79	0.00	0.00	0.16	0.79
CAMKK2	Pool 03	16.82	36	0.18	1.10	0.11	0.68	0.10	0.61	0.24	1.43	0.00	0.00	0.24	1.43
CAMKK2	Pool 07	19.36	36	0.21	1.07	0.03	0.14	0.09	0.45	0.23	1.17	0.00	0.00	0.23	1.17
KLK3	Pool 05	14.87	36	0.09	0.57	0.24	1.60	0.00	0.00	0.25	1.70	0.13	0.87	0.28	1.91
KLK3	Pool 08	15.78	36	0.11	0.72	0.09	0.57	0.05	0.29	0.15	0.96	0.11	0.70	0.19	1.19
KLK3	Pool 04	14.20	36	0.41	2.86	0.26	1.86	0.00	0.00	0.48	3.41	0.00	0.00	0.48	3.41
KLK3	Pool 01	14.37	36	0.15	1.07	0.04	0.27	0.00	0.00	0.16	1.11	0.16	1.08	0.22	1.55
KLK3	Pool 07	16.27	36	0.28	1.72	0.00	0.00	0.00	0.00	0.28	1.72	0.15	0.92	0.32	1.95
KLK3	Pool 06	15.51	36	0.16	1.01	0.23	1.51	0.00	0.00	0.28	1.82	0.12	0.75	0.31	1.97
KLK3	Pool 02	13.30	36	0.18	1.36	0.28	2.09	0.00	0.00	0.33	2.49	0.14	1.02	0.36	2.69
KLK3	Pool 03	12.72	36	0.17	1.30	0.25	1.95	0.00	0.00	0.30	2.34	0.11	0.89	0.32	2.50
OR51E2	Pool 04	18.42	36	0.34	1.86	0.08	0.45	0.00	0.00	0.35	1.91	0.20	1.08	0.40	2.20
OR51E2	Pool 01	18.79	36	0.12	0.64	0.10	0.55	0.00	0.00	0.16	0.84	0.23	1.21	0.28	1.48
OR51E2	Pool 05	19.19	36	0.18	0.95	0.08	0.39	0.00	0.00	0.20	1.03	0.26	1.34	0.32	1.69
OR51E2	Pool 07	21.14	36	0.17	0.81	0.19	0.92	0.00	0.00	0.26	1.22	0.29	1.37	0.39	1.84
OR51E2	Pool 08	21.06	36	0.21	0.97	0.17	0.83	0.00	0.00	0.27	1.28	0.28	1.35	0.39	1.86
OR51E2	Pool 02	18.26	36	0.17	0.94	0.02	0.08	0.00	0.00	0.17	0.95	0.28	1.55	0.33	1.82
OR51E2	Pool 06	21.39	36	0.22	1.02	0.19	0.91	0.00	0.00	0.29	1.37	0.23	1.09	0.37	1.75
OR51E2	Pool 03	17.62	36	0.16	0.93	0.14	0.80	0.00	0.00	0.22	1.23	0.24	1.39	0.33	1.85
PCA3	Pool 05	17.85	36	0.12	0.68	0.20	1.09	0.00	0.00	0.23	1.29	0.09	0.52	0.25	1.39
PCA3	Pool 07	19.87	36	0.17	0.85	0.13	0.67	0.08	0.39	0.23	1.15	0.07	0.33	0.24	1.20
PCA3	Pool 06	19.42	36	0.11	0.58	0.17	0.87	0.00	0.00	0.20	1.05	0.13	0.65	0.24	1.23
PCA3	Pool 08	20.37	36	0.15	0.75	0.15	0.74	0.00	0.00	0.21	1.05	0.07	0.36	0.23	1.11
PCA3	Pool 04	15.85	36	0.40	2.50	0.00	0.00	0.16	1.01	0.43	2.69	0.00	0.00	0.43	2.69
PCA3	Pool 01	18.16	36	0.08	0.46	0.08	0.46	0.13	0.74	0.18	0.99	0.14	0.75	0.22	1.24
PCA3	Pool 03	16.04	36	0.18	1.11	0.23	1.41	0.00	0.00	0.29	1.80	0.09	0.55	0.30	1.88
PCA3	Pool 02	16.55	36	0.10	0.60	0.16	0.97	0.02	0.14	0.19	1.15	0.11	0.65	0.22	1.32
PCAT14	Pool 06	21.62	36	0.13	0.60	0.15	0.72	0.00	0.00	0.20	0.93	0.06	0.29	0.21	0.98
PCAT14	Pool 02	20.15	36	0.12	0.61	0.19	0.93	0.04	0.21	0.23	1.14	0.05	0.24	0.23	1.16
PCAT14	Pool 08	23.48	36	0.21	0.90	0.00	0.00	0.20	0.85	0.29	1.24	0.00	0.00	0.29	1.24
PCAT14	Pool 01	20.86	36	0.18	0.85	0.02	0.12	0.13	0.64	0.22	1.07	0.00	0.00	0.22	1.07
PCAT14	Pool 07	22.75	36	0.24	1.04	0.10	0.44	0.12	0.51	0.28	1.24	0.00	0.00	0.28	1.24
PCAT14	Pool 03	19.61	36	0.17	0.85	0.20	0.99	0.06	0.28	0.26	1.34	0.00	0.00	0.26	1.34
PCAT14	Pool 05	23.57	36	0.35	1.48	0.18	0.78	0.29	1.23	0.49	2.07	0.00	0.00	0.49	2.07
PCAT14	Pool 04	18.15	36	0.29	1.61	0.17	0.95	0.11	0.61	0.36	1.97	0.00	0.00	0.36	1.97
SCHLAP1	Pool 02	21.65	36	0.13	0.58	0.10	0.47	0.18	0.81	0.24	1.11	0.10	0.44	0.26	1.19
SCHLAP1	Pool 05	23.04	36	0.24	1.03	0.00	0.00	0.31	1.33	0.39	1.68	0.00	0.00	0.39	1.68
SCHLAP1	Pool 03	20.61	36	0.16	0.78	0.19	0.93	0.00	0.00	0.25	1.21	0.10	0.50	0.27	1.31
SCHLAP1	Pool 07	24.19	36	0.41	1.70	0.00	0.00	0.38	1.55	0.56	2.30	0.00	0.00	0.56	2.30
SCHLAP1	Pool 04	21.28	36	0.47	2.20	0.00	0.00	0.30	1.42	0.56	2.62	0.00	0.00	0.56	2.62
SCHLAP1	Pool 08	23.14	36	0.31	1.35	0.12	0.53	0.00	0.00	0.33	1.44	0.17	0.75	0.38	1.63
SCHLAP1	Pool 06	22.80	36	0.34	1.47	0.21	0.94	0.00	0.00	0.40	1.75	0.06	0.27	0.40	1.77
SCHLAP1	Pool 01	21.61	36	0.20	0.92	0.08	0.36	0.20	0.92	0.29	1.35	0.07	0.31	0.30	1.38
SPON2	Pool 02	18.94	36	0.20	1.03	0.07	0.37	0.13	0.68	0.24	1.29	0.09	0.47	0.26	1.37
SPON2	Pool 04	19.30	36	0.53	2.72	0.26	1.32	0.22	1.15	0.63	3.24	0.00	0.00	0.63	3.24
SPON2	Pool 06	20.90	36	0.28	1.34	0.18	0.86	0.14	0.67	0.36	1.73	0.00	0.00	0.36	1.73
SPON2	Pool 03	18.66	36	0.31	1.66	0.00	0.00	0.00	0.00	0.31	1.66	0.00	0.00	0.31	1.66
SPON2	Pool 01	19.36	36	0.14	0.74	0.00	0.00	0.19	0.98	0.24	1.22	0.10	0.54	0.26	1.34
SPON2	Pool 08	21.77	36	0.28	1.27	0.00	0.00	0.00	0.00	0.28	1.27	0.05	0.25	0.28	1.30
SPON2	Pool 05	19.11	36	0.15	0.77	0.38	1.97	0.00	0.00	0.40	2.12	0.00	0.00	0.40	2.12
SPON2	Pool 07	21.20	36	0.34	1.58	0.00	0.00	0.18	0.84	0.38	1.79	0.05	0.24	0.38	1.81
T2ERG	Pool 06	28.00	36	0.00	0.00	0.00	0.00	0.00	0.00	0.00	0.00	0.00	0.00	0.00	0.00
T2ERG	Pool 08	27.91	36	0.35	1.24	0.00	0.00	0.00	0.00	0.35	1.24	0.00	0.00	0.35	1.24
T2ERG	Pool 02	23.51	36	0.30	1.28	0.22	0.92	0.30	1.26	0.47	2.01	0.00	0.00	0.47	2.01
T2ERG	Pool 03	23.74	36	0.47	2.00	0.17	0.73	0.34	1.43	0.61	2.56	0.00	0.00	0.61	2.56
T2ERG	Pool 05	27.98	36	0.12	0.43	0.00	0.00	0.00	0.00	0.12	0.43	0.00	0.00	0.12	0.43
T2ERG	Pool 07	28.00	36	0.00	0.00	0.00	0.00	0.00	0.00	0.00	0.00	0.00	0.00	0.00	0.00
T2ERG	Pool 04	28.00	36	0.00	0.00	0.00	0.00	0.00	0.00	0.00	0.00	0.00	0.00	0.00	0.00
T2ERG	Pool 01	23.30	36	0.33	1.42	0.00	0.00	0.24	1.04	0.41	1.76	0.00	0.00	0.41	1.76
TFF3	Pool 01	18.92	36	0.17	0.90	0.00	0.00	0.12	0.66	0.21	1.12	0.13	0.71	0.25	1.32
TFF3	Pool 07	20.72	36	0.24	1.14	0.00	0.00	0.08	0.37	0.25	1.20	0.13	0.63	0.28	1.36
TFF3	Pool 06	21.46	36	0.22	1.03	0.17	0.81	0.00	0.00	0.28	1.31	0.13	0.60	0.31	1.44
TFF3	Pool 02	18.38	36	0.16	0.84	0.09	0.51	0.12	0.63	0.22	1.17	0.13	0.73	0.25	1.38
TFF3	Pool 03	17.33	36	0.67	3.84	0.00	0.00	0.18	1.06	0.69	3.98	0.17	0.97	0.71	4.10
TFF3	Pool 05	19.29	36	0.14	0.73	0.20	1.04	0.07	0.34	0.25	1.31	0.09	0.47	0.27	1.40
TFF3	Pool 04	18.54	36	0.42	2.25	0.16	0.87	0.21	1.16	0.50	2.67	0.00	0.00	0.50	2.67
TFF3	Pool 08	21.34	36	0.18	0.85	0.11	0.50	0.09	0.44	0.23	1.08	0.13	0.61	0.27	1.24
TRGV9	Pool 04	14.67	36	0.53	3.60	0.25	1.70	0.00	0.00	0.58	3.98	0.00	0.00	0.58	3.98
TRGV9	Pool 06	16.49	36	0.21	1.28	0.27	1.62	0.00	0.00	0.34	2.07	0.12	0.75	0.36	2.20
TRGV9	Pool 05	16.10	36	0.16	1.00	0.28	1.76	0.00	0.00	0.33	2.02	0.00	0.00	0.33	2.02
TRGV9	Pool 03	13.63	36	0.21	1.55	0.20	1.49	0.00	0.00	0.29	2.15	0.09	0.67	0.31	2.25
TRGV9	Pool 02	14.36	36	0.18	1.27	0.28	1.97	0.00	0.00	0.34	2.35	0.16	1.12	0.37	2.60
TRGV9	Pool 01	14.76	36	0.12	0.81	0.00	0.00	0.03	0.22	0.12	0.84	0.10	0.67	0.16	1.08
TRGV9	Pool 07	17.49	36	0.37	2.09	0.00	0.00	0.14	0.83	0.39	2.25	0.08	0.48	0.40	2.30
TRGV9	Pool 08	16.54	36	0.13	0.76	0.12	0.73	0.11	0.69	0.21	1.26	0.11	0.64	0.23	1.41

**Table 6:** MPS2-AS risk scores for between-reagent-lot, within-lab reproducibility and variance contributions from between-day, between-run and between-replicate (repeatability) sources.

				Repeatability		Between-Run		Between-Day		Within-Lab		Between-Lot		Reproducibility		
	Pool ID	Mean	N	SD	%CV	SD	%CV	SD	%CV	SD	%CV	SD	%CV	SD	%CV	Psad
GG1-2	Pool 01	36.59	36	3.02	8.25	1.84	5.02	0.69	1.87	3.60	9.83	0.00	0.00	3.60	9.83	0.10
GG1-2	Pool 02	55.59	36	2.37	4.27	2.68	4.83	1.43	2.57	3.86	6.94	0.00	0.00	3.86	6.94	0.30
GG1-2	Pool 03	65.01	36	3.73	5.74	0.98	1.51	1.59	2.45	4.17	6.42	0.00	0.00	4.17	6.42	0.34
GG1-2	Pool 04	78.52	36	1.60	2.04	0.62	0.79	0.62	0.79	1.83	2.33	0.00	0.00	1.83	2.33	0.40
GG1-2	Pool 05	8.47	36	0.37	4.35	0.31	3.70	0.38	4.47	0.61	7.25	0.00	0.00	0.61	7.25	0.02
GG1-2	Pool 06	51.21	36	2.65	5.18	2.59	5.05	1.77	3.46	4.11	8.02	0.00	0.00	4.11	8.02	0.32
GG1-2	Pool 07	63.71	36	3.11	4.88	0.00	0.00	1.93	3.03	3.66	5.74	0.00	0.00	3.66	5.74	0.40
GG1-2	Pool 08	8.67	36	0.52	5.99	0.47	5.47	0.10	1.21	0.71	8.20	0.00	0.00	0.71	8.20	0.04
GG1-3	Pool 01	11.01	36	0.57	5.19	0.00	0.00	0.39	3.54	0.69	6.29	0.00	0.00	0.69	6.29	0.10
GG1-3	Pool 02	26.66	36	1.00	3.74	1.18	4.43	0.00	0.00	1.55	5.80	0.00	0.00	1.55	5.80	0.30
GG1-3	Pool 03	31.65	36	0.73	2.32	0.88	2.77	0.28	0.89	1.18	3.72	0.00	0.00	1.18	3.72	0.34
GG1-3	Pool 04	55.94	36	1.26	2.26	0.90	1.60	0.00	0.00	1.55	2.77	0.25	0.44	1.57	2.81	0.40
GG1-3	Pool 05	6.36	36	0.29	4.62	0.00	0.00	0.24	3.79	0.38	5.97	0.00	0.00	0.38	5.97	0.02
GG1-3	Pool 06	26.00	36	0.81	3.13	1.31	5.04	0.46	1.76	1.61	6.19	0.00	0.00	1.61	6.19	0.32
GG1-3	Pool 07	38.66	36	1.49	3.85	0.00	0.00	1.18	3.05	1.90	4.92	0.00	0.00	1.90	4.92	0.40
GG1-3	Pool 08	5.85	36	0.20	3.41	0.16	2.76	0.17	2.96	0.31	5.29	0.04	0.76	0.31	5.35	0.04

## Data Availability

The raw data supporting the conclusions of this article will be made available by the authors upon reasonable request.

## Abbreviations

The following abbreviations are used in this manuscript:

ANOVA	Analysis of variance
AS	Active surveillance
CAMKK2	Calcium/calmodulin-dependent protein kinase kinase 2
cDNA	Complementary DNA
CLSI	Clinical and Laboratory Standards Institute
CV	Coefficient of variation
DRE	Digital rectal examination
GG	Grade Group
KLK3	Kallikrein-related peptidase 3
mpMRI	Multiparametric magnetic resonance imaging
MPS2	MyProstateScore 2.0
MPS2-AS	MyProstateScore 2.0—Active Surveillance
NCCN	National Comprehensive Cancer Network
NPV	Negative predictive value
OR51E2	Olfactory receptor family 51 subfamily E member 2
PCa	Prostate cancer
PCA3	Prostate cancer associated 3
PCAT14	Prostate cancer associated transcript 14
PCR	Polymerase chain reaction
PSA	Prostate-specific antigen
PSAD	Prostate-specific antigen density
qPCR	Quantitative polymerase chain reaction
RNA	Ribonucleic acid
RT-PCR	Real-time polymerase chain reaction
SCHLAP1	SWI/SNF complex antagonist associated with prostate cancer 1
SD	Standard deviation
SPON2	Spondin 2
T2ERG	TMPRSS2:ERG fusion
TFF3	Trefoil factor 3
TMPRSS2:ERG	Transmembrane serine protease 2: ETS-related gene fusion
TRGV9	T-cell receptor gamma variable 9
